# Response to: Comment on “Prevalence and Risk Factors for Diabetic Lower Limb Amputation: A Clinic-Based Case Control Study”

**DOI:** 10.1155/2018/1908140

**Published:** 2018-02-20

**Authors:** Beverly T. Rodrigues, Venkat N. Vangaveti, Usman H. Malabu

**Affiliations:** Department of Diabetes and Endocrinology, The Townsville Hospital and College of Medicine and Dentistry, James Cook University, 100 Angus Smith Drive, Douglas, QLD 4814, Australia

The observations by Bakhtiyari and Mansournia on our study [1] were received with great interest. We believe our study was consistent with a case-control format [2]. In particular, our target population was selected from subjects diagnosed with diabetic foot ulcer (DFU) at the local high-risk foot clinic, effectively fulfilling the criteria of *control* (DFU without amputations) and *case* (DFU with amputation), as previously described [3]. The identified risk factors were computed amongst those with and without limb amputations. We do not believe our study was cross-sectional, being retrospective in design, and it was not conducted at a specific point in time rather for a period from January 2011 to December 2013; both are in agreement with the definition of a case-control study [4]. The prevalence of diabetic limb amputation quoted in our study was for our high-risk diabetic foot clinic and not for the general population of North Eastern Australia which was clearly outlined in the title as *clinic-based* [5]. Interestingly, in line with Bakhtiyari's and Mansournia's observation of a case-control study, our sampling was based on the outcome (amputation) and was known in advance prior to conducting the study [3]. Furthermore, in keeping with a case-control study, we did not undertake propensity matching since cases and controls were outcomes rather than exposures [4]. With respect to our discussion on the prevalence of diabetic limb amputation, comparing clinic-based and non-clinic-based studies, the point was noted but we clearly stated our study was similar to those reported by others [6]. Overall, we do not think the title of our article was confusing since it tallied with the methodology and content of the article.
